# Abdominal emergencies in the geriatric patient

**DOI:** 10.1186/s12245-014-0043-2

**Published:** 2014-10-21

**Authors:** Ryan Spangler, Thuy Van Pham, Danya Khoujah, Joseph P Martinez

**Affiliations:** 1 Department of Emergency Medicine, University of Maryland School of Medicine, 110 South Paca Street, 6th Floor, Suite 200, Baltimore 21201, MD, USA

**Keywords:** Abdominal pain, Mesenteric ischemia, Appendicitis, Elderly, Abdominal aortic aneurysm

## Abstract

Abdominal pain is one of the most frequent reasons that elderly people visit the emergency department (ED). In this article, we review the deadliest causes of abdominal pain in this population, including mesenteric ischemia, abdominal aortic aneurysm, and appendicitis and potentially lethal non-abdominal causes. We also highlight the pitfalls in diagnosing, or rather *misdiagnosing*, these clinical entities.

## 1 Review

### 1.1 Introduction

The world's population is increasing, and the elderly represent its fastest growing segment. The number of emergency department (ED) visits for the geriatric population is also increasing. Providing care to elderly patients presents its own unique set of challenges. This is especially true for elderly patients presenting with acute abdominal pain. This subset of patients is at extremely high risk, with a mortality rate approaching 10% [1]. They also consume a tremendous amount of ED resources, requiring laboratory testing, imaging, and consultant services at significantly higher rates than younger patients. Elderly patients with acute abdominal pain present diagnostic challenges as well. Their distinctive physiology leads to atypical presentations, with delayed symptoms, less predictable alterations in vital signs in response to disease, and markedly unreliable physical examinations. The unwary practitioner can often be falsely reassured by the patient's seemingly innocuous appearance and deceptively normal laboratory values. In this paper, we highlight some of the unique ways that otherwise straightforward disease processes present in the elderly and present strategies for their management.

#### 1.1.1 Vascular disorders

Being the most time sensitive of all diagnoses, vascular disorders should be considered early in the course of any elderly patient presenting with acute abdominal pain.

##### Acute mesenteric ischemia

Acute mesenteric ischemia (AMI) is a nonspecific term encompassing disease processes that result in ischemic damage due to decreased blood flow from the mesenteric vascular system (Table 1). Although the overall incidence of mesenteric ischemia is low in the ED population, it is more common and is acutely life-threatening, with mortality estimates above 50% [2]. Many of the specific risk factors for AMI increase in prevalence in older populations.

**Table 1:** Mesenteric ischemia

**Types**	**Risk factors**	**Presentations**
SMA embolus	Atrial fibrillation, dilated cardiomyopathy, arrhythmia, valvular disease, previous embolic events	Pain out of proportion to physical exam findings; nausea, vomiting, diarrhea
SMA thrombosis	Atherosclerosis, smoking	Similar to SMA embolus, but my have long-standing postprandial abdominal pain or ‘intestinal angina’
SMV thrombosis	Hypercoagulable state, oral contraceptive use	Less severe pain than arterial disease; more indolent course
NOMI	Low-flow state/ICU patients: sepsis, hypotension, severe volume depletion, dialysis; cocaine users; trauma patients	Nonreproducible abdominal pain; unexplained GI bleeding in ICU patients; abdominal pain after dialysis

**Figure 1:**
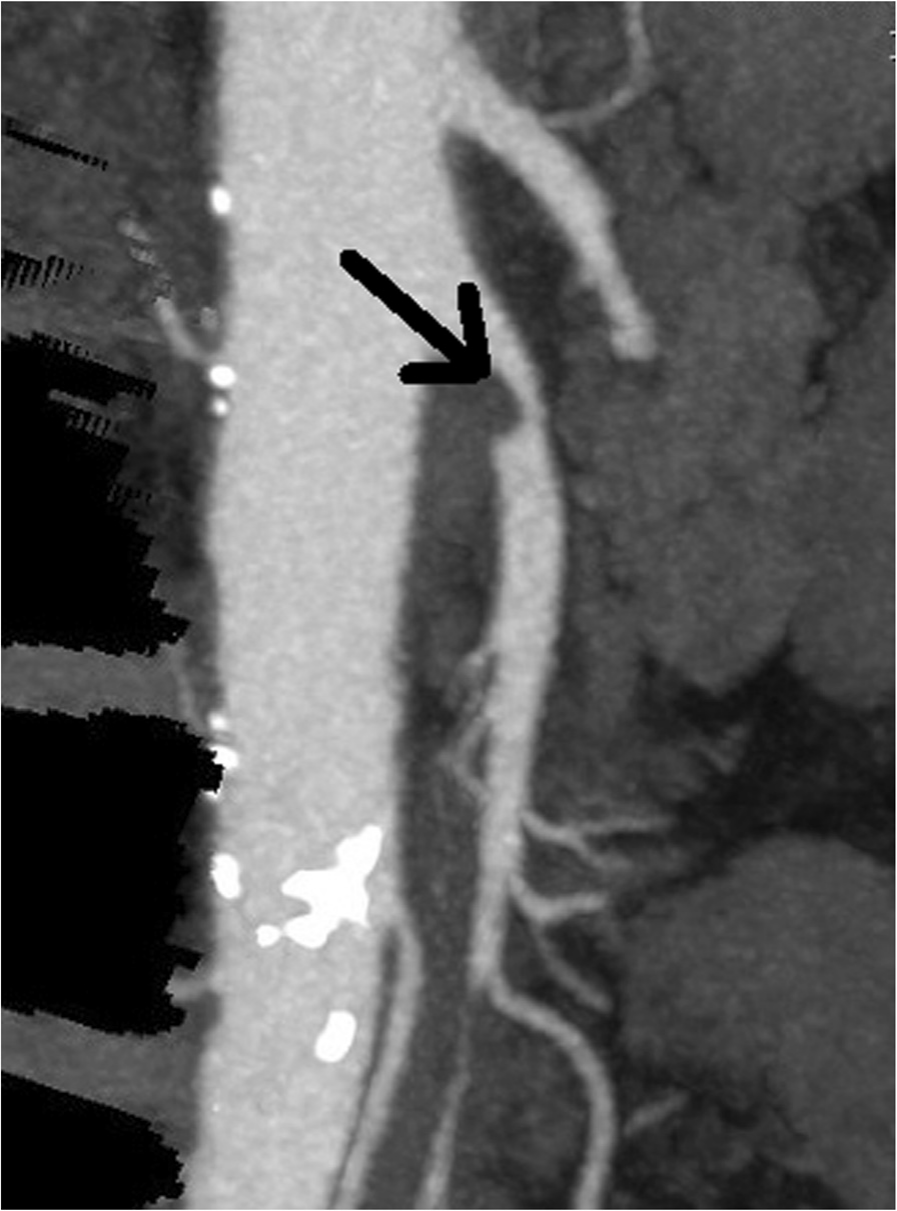
CT angiogram demonstrating stenosis of the superior mesenteric artery.

Superior mesenteric artery (SMA) embolus is the most common variety [3]. Patients at highest risk for this type of mesenteric ischemia have a cardiac source of emboli, such as atrial fibrillation, dilated cardiomyopathy, arrhythmia, and valvular disease [4]. Approximately one-third of these patients have a history of an embolic event [5]. Thrombosis of the SMA, about 15% of AMI cases, is found in patients with typical atherosclerosis risk factors. Deposition of plaque at the origin of the SMA can lead to flow-limiting stenosis (Figure 1). Patients with this condition may have a history of long-standing post-prandial abdominal pain or ‘intestinal angina,’ a sign of chronic mesenteric ischemia [6]. Plaque rupture can occlude the SMA, leading to acute SMA thrombosis.

Superior mesenteric vein (SMV) thrombosis, often caused by a hypercoagulable state, is present in 5% to 15% of cases of AMI. Patients with this condition are usually much younger than patients with SMA embolus. Half of these patients have a personal or family history of venous thromboembolism. Similar to SMA thrombosis, this course can be indolent and nonspecific [7].

Non-occlusive mesenteric ischemia (NOMI) develops as the result of a low-flow state with vasospasm of the branches of the SMA, rather than acute occlusion. NOMI can develop in patients who are hypotensive, on vasopressors, severely volume depleted, or on dialysis. Generally more common in critically ill patients, it may occur acutely in situations such as trauma or cocaine abuse. NOMI has a very high mortality rate, likely due to the combination of comorbidities and the difficulty in making this diagnosis.

Clinicians in the ED must be aware of a patient's risk factors for AMI and maintain a high level of suspicion for this disease. Classically, the patient presents with nonreproducible abdominal pain, commonly referred to as ‘pain out of proportion to exam findings.’ This reflects the visceral, rather than a peritoneal, origin of the pain [8]. However, some patients might present initially with vomiting and diarrhea, complaints of intermittent abdominal pain when eating, or other more subtle complaints. Traditional teaching is that laboratory tests, such as measurement of the lactic acid level, can be helpful in identifying patients at greater risk; however, there is no specific lab test for mesenteric ischemia. Lactate levels could be normal in those who present early; elevation is often a late finding [9]. Surgical consult and appropriate imaging early in the course have been shown to improve outcomes, as this is a time-sensitive diagnosis. Angiography is the traditional test of choice and has been shown to decrease the risk of mortality if performed early [7]. Multidetector-row computed tomography (CT) has demonstrated good accuracy in cases of AMI. It has the advantages of being more readily available and less invasive than angiography. It can also elucidate other causes of severe abdominal pain [10].

##### Abdominal aortic aneurysm

Abdominal aortic aneurysm (AAA) is a disease found almost exclusively in the elderly, and rupture of an AAA carries an extremely high mortality rate [11]. AAA can be a straightforward diagnosis in classic presentations but extraordinarily challenging in atypical cases. It can present similarly to more benign diagnoses such as renal colic or musculoskeletal back pain, meaning it must be considered early in the course of a wide variety of patient complaints. Bedside ultrasound and CT are rapid, reliable, noninvasive tests that can assist in making this diagnosis.

The classic presentation of ruptured AAA is hypotension, abdominal pain, and a pulsatile abdominal mass. While classic, this combination is found in less than half of cases [12]. Hypotension might be transient and could have resolved if the bleeding is retroperitoneal and has tamponaded temporarily. Rupture can also present with isolated back rather than abdominal pain [12]. A urine dipstick could be positive for blood as a result of irritation of the ureter by the AAA. A frequent misdiagnosis in patients with back pain and microscopic hematuria is renal colic. Extreme caution must be taken before diagnosing an elderly individual with new renal colic, musculoskeletal back pain, or even syncope without considering ruptured AAA [13].

**Figure 2:**
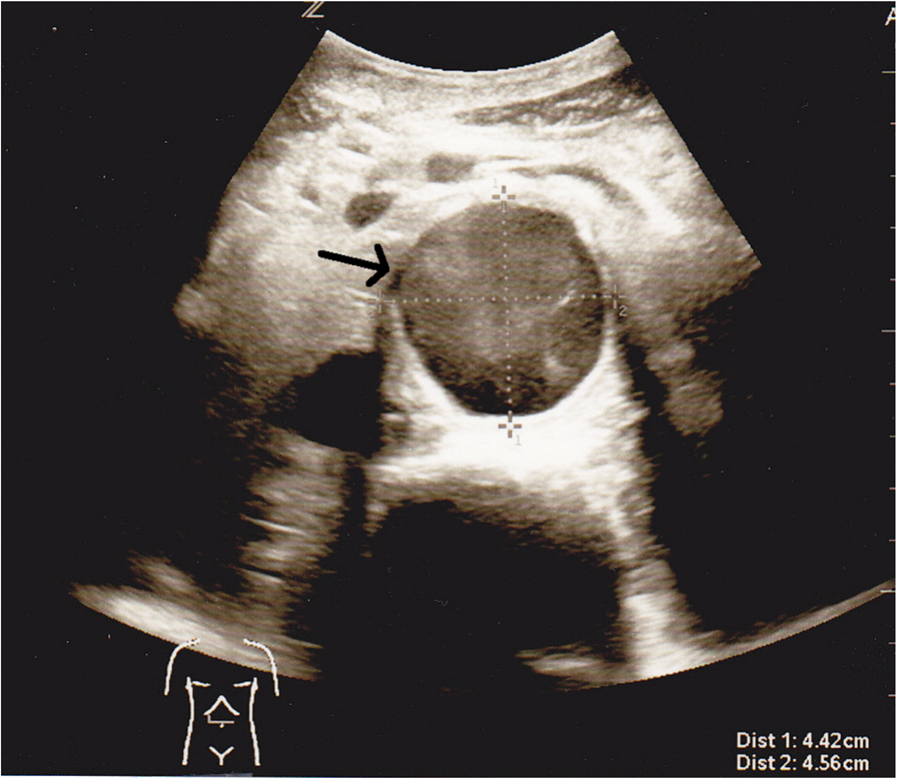
Ultrasound image diagnostic for abdominal aortic aneurysm.

Once the diagnosis of AAA is entertained, it can be excluded rapidly and reliably with basic imaging. The fastest, least expensive, and least invasive technique is bedside ultrasound (Figure 2). Even novice users can be trained to identify an AAA accurately and effectively identify using this modality [14],[15]. For many physicians, ultrasound is rapidly becoming the bedside tool of choice, and AAA is one diagnosis that supports this movement. CT is very accurate at detecting not only the AAA but also the presence of retroperitoneal hemorrhage (an area where ultrasound falls short). Even a noncontrast CT scan can accurately identify the presence of an AAA and any associated hemorrhage without the risk of contrast nephropathy, allergic reactions, or extra time needed to obtain contrast studies [16].

#### 1.1.2 Intestinal disorders

##### Bowel obstruction

Small bowel obstruction (SBO) in the elderly is the second most commonly missed surgical emergency, after appendicitis [17]. As in young patients, hernias and adhesions are the leading cause of SBO in the elderly. Causes seen uniquely in the elderly include neoplasm and gallstone ileus (Table 2). Although the presentation of SBO is similar in the elderly, the mortality rate is much higher [18].

**Table 2:** Causes of bowel obstruction

**Small bowel obstruction**	**Large bowel obstruction**
Hernias/adhesion	Neoplasm/mass
Neoplasm/mass	Diverticulitis
Gallstones	Volvulus

**Figure 3:**
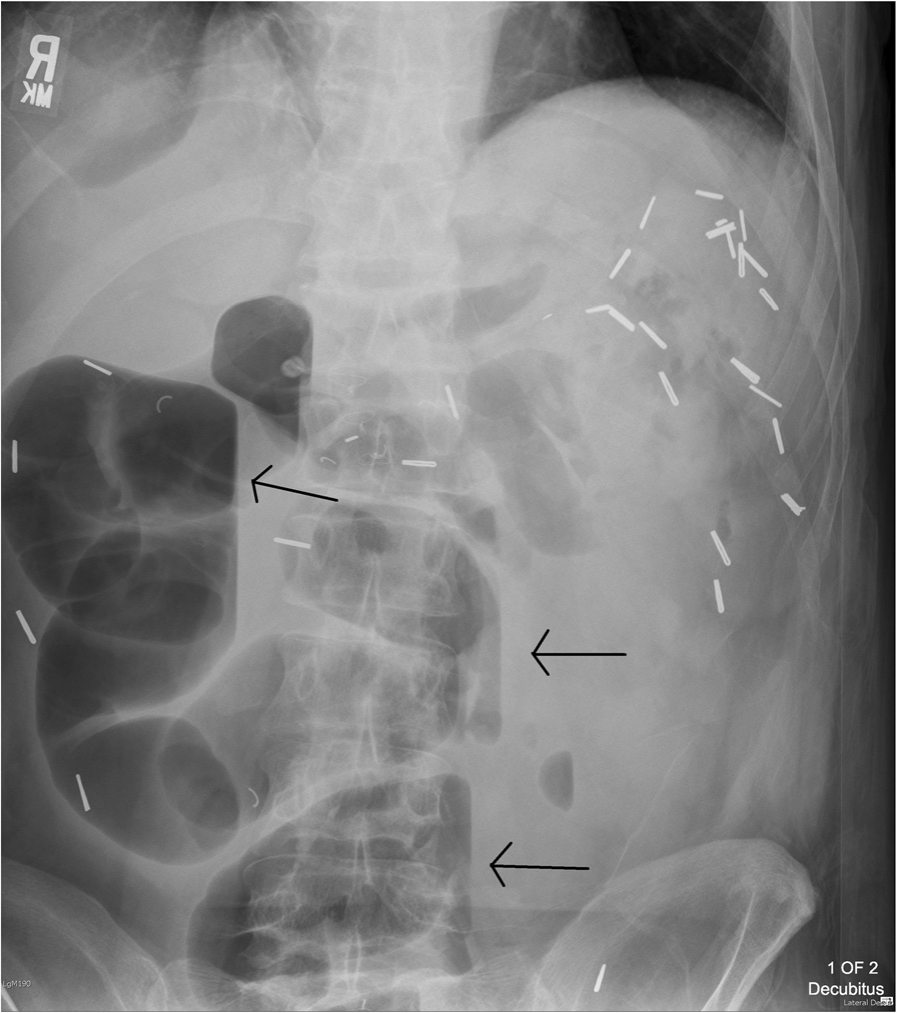
**Left lateral decubitus radiograph demonstrating air-fluid levels.** Incidental surgical clips from prior bowel resection are also noted.

Plain radiographs of the abdomen might show evidence of SBO, such as dilated bowel and air-fluid levels (Figure 3). However, the absence of these findings does not rule out obstruction. CT has higher sensitivity for detection of SBO and might identify the cause and location [19].

**Figure 4:**
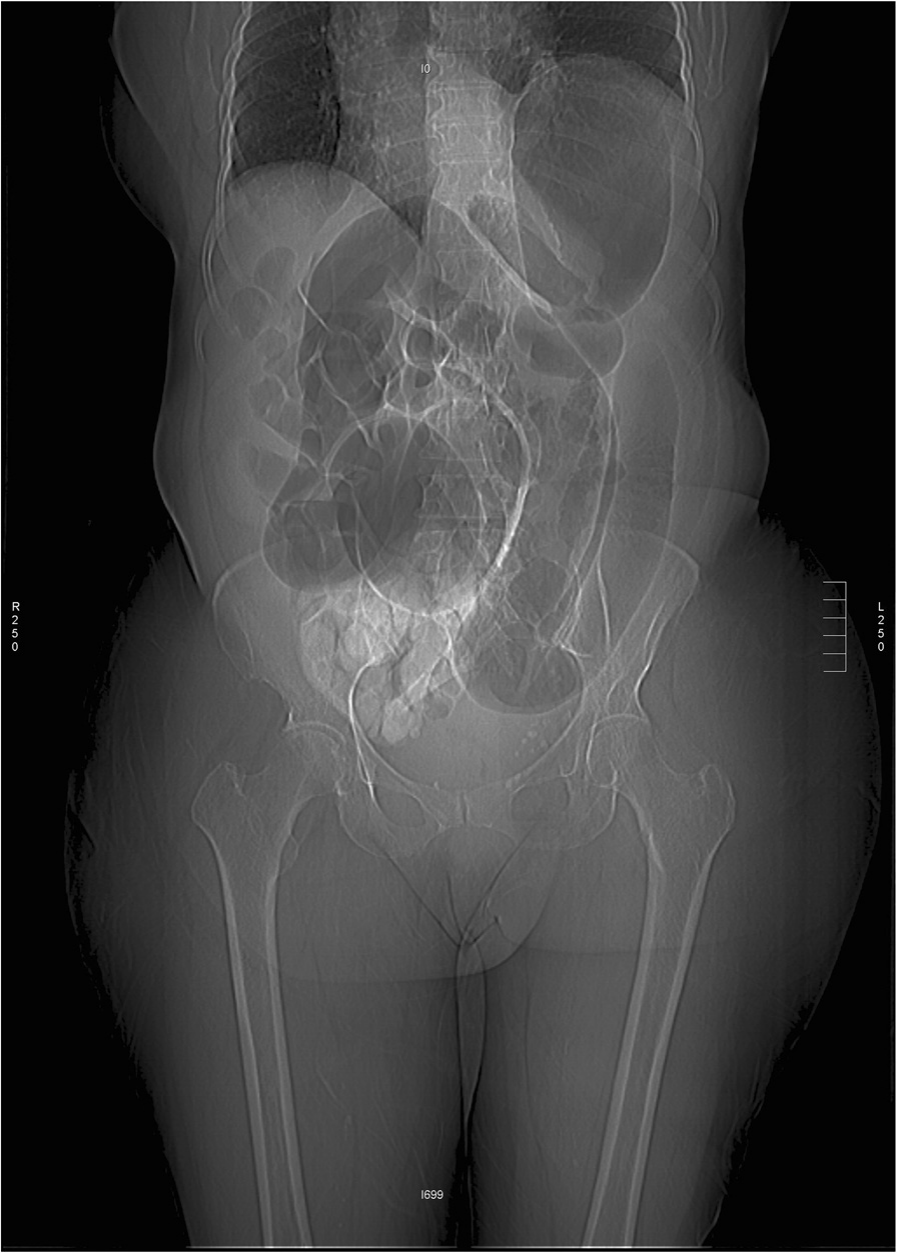
Radiograph demonstrating sigmoid volvulus.

Large bowel obstructions are much more common in the elderly because of the increased incidence of cancer and diverticulitis in this age group. Though patients classically present with abdominal pain, constipation, and vomiting, nearly half do not have vomiting or constipation. Many complain of diarrhea [20]. Sigmoid and cecal volvuli also cause large bowel obstruction. Cecal volvulus tends to present acutely in a younger population and usually requires emergent surgery. Sigmoid volvulus should be suspected in the chronically ill, debilitated patient and is often of slower onset [21] (Figure 4). Initial management can consist of nonoperative decompression through sigmoidoscopy or barium enema. However, because of the high incidence of recurrence, definitive surgery in a delayed manner is often required.

##### Diverticular disease

The prevalence of diverticular disease, or diverticulosis, rises dramatically in the elderly, reaching nearly 80% in people over the age of 85 [22]. Colonic diverticulae are usually asymptomatic, but they can become inflamed (diverticulitis) or bleed.

Diverticulitis occurs in 10% to 20% of patients with diverticular disease, and it is recurrent in 25% of cases [23]. Classically, patients present with fever, nausea, change in bowel regimen (constipation, diarrhea, or tenesmus), and left lower quadrant (LLQ) pain. They may have a tender LLQ mass and leukocytosis as well. However, older patients might present atypically. Almost half are afebrile and many have a normal white blood cell count [24]. Thirty percent do not have abdominal tenderness on exam [25]. In fact, nearly half of all cases of diverticulitis are misdiagnosed initially [26]. Some of the more common misdiagnoses include urinary tract infection and renal colic, as there is a high incidence of concomitant urinary symptoms. When the right colon is predominantly involved, clinicians might suspect appendicitis. Therefore, the liberal use of CT is recommended, as it is both highly sensitive and specific for this disease, whether or not contrast is used [27]. In addition, it allows diagnosis of complications of diverticulitis as well as other disease processes masquerading as it.

Diverticulitis might be complicated by the formation of an abscess or fistula, bowel obstruction, free perforation, or the development of sepsis. The elderly are at increased risk of these complications and have an increased mortality rate when they develop [28]. The complications are managed surgically or through interventional radiology, similar to the approach in younger patients.

Patients who are well appearing, have no comorbidities, and have access to good follow-up care may be managed as outpatients, with a low-residue diet and oral antibiotics effective against gram-negative organisms and anaerobes for 7 to 10 days. Most elderly patients require admission for intravenous broad-spectrum antibiotics, bowel rest, and rehydration, in addition to analgesics and anti-emetics as needed. Elderly patients with diverticulitis should have a colonoscopy or sigmoidoscopy performed 4 to 6 weeks after resolution of symptoms to exclude an underlying carcinoma, which is present in up to 15% [29].

Bleeding occurs in 15% of patients with diverticulosis. It is the most common cause of lower gastrointestinal bleeding in the elderly. The bleeding is usually mild, but occasionally it is massive. Bleeding ceases spontaneously in 90%, and rebleeding recurs in up to 25%. Multiple risk factors have been associated with bleeding, such as hypertension, anticoagulation, diabetes mellitus, and ischemic heart disease [30]. Diverticular bleeding should be managed initially as any other cause of lower GI bleeding, keeping in mind the importance of early resuscitation and aggressive management and monitoring, given the elderly patient's decreased physiologic reserve.

##### Appendicitis

Appendicitis is the most common abdominal surgical emergency in the general population and the third most common indication for abdominal surgery in the elderly patient [31],[32]. The incidence of appendicitis is increasing in the elderly population secondary to the increasing life expectancy [31]. Although the overall incidence is lower in the elderly population compared with the general population, the mortality rate is four to eight times higher [31]-[33]. Up to half of all deaths from appendicitis occur in elderly patients [34]. The high mortality rate is attributed to delayed and atypical presentations leading to frequent misdiagnosis.

**Figure 5:**
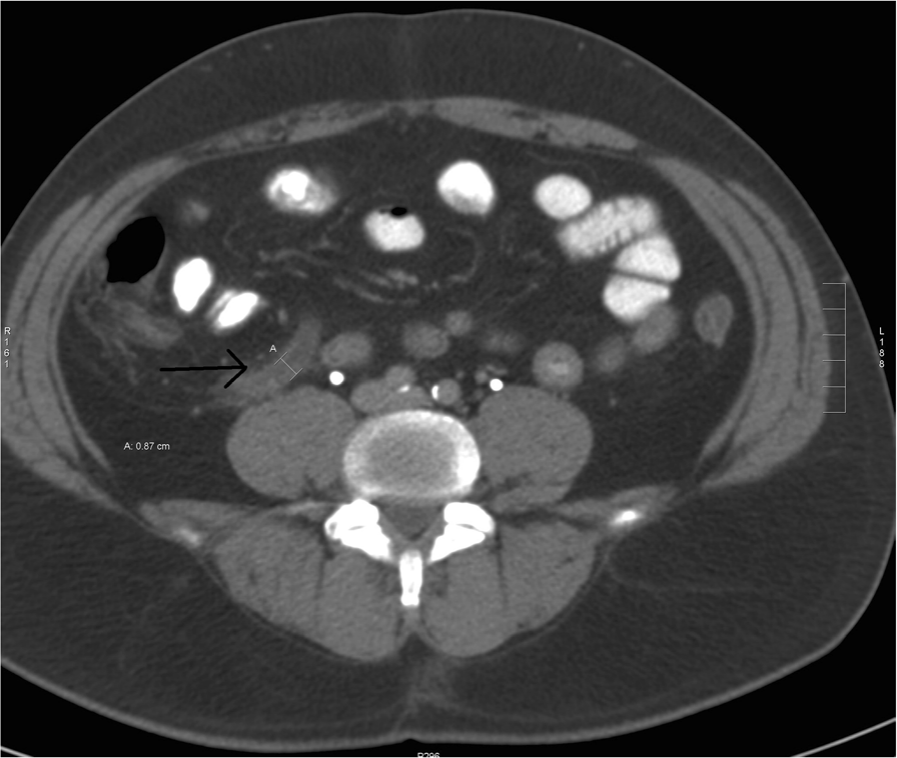
CT scan showing an inflamed appendix.

Despite the advances in modern medicine, appendicitis is still misdiagnosed 54% of the time in the elderly patient population [35]. Half of the patients who are misdiagnosed have bowel perforation by the time of surgery [35]. One-fifth of all elderly patients with appendicitis present after 3 days of symptoms and another 5% to 10% of patients present after 1 week of symptoms [36]. Less than one-third of patients have fever, anorexia, right lower quadrant pain, or leukocytosis. One-quarter of patients have no right lower quadrant pain at all [35],[37],[38]. Though multiple scoring systems have been developed to risk-stratify patients with suspected appendicitis, they have not demonstrated sufficient discriminatory or predictive ability to be used in the elderly population [31]. High clinical suspicion and liberal use of CT scanning in elderly patients is necessary to make this diagnosis in a timely fashion (Figure 5).

#### 1.1.3 Miscellaneous causes of abdominal pain

##### Peptic ulcer disease

Peptic ulcer disease (PUD) is a common and often undiagnosed disease among elderly patients. Approximately half of patients over the age of 60 with PUD initially present with a complication, most often perforation [39],[40]. Other complications include hemorrhage, gastric outlet obstruction, and erosion into an adjacent structure [40]. It has been shown that up to 35% of people over the age of 60 with endoscopically proven PUD did not have any abdominal pain, in contrast to only 8% of patients under the age of 60 [40]-[42].

Elderly patients with PUD have a higher mortality rate than the general population [43],[44]. They are more likely to require blood transfusion, to undergo surgery to control bleeding, and to rebleed [45]. The mortality rate associated with perforation in the elderly is 30% compared with 10% in the general population. If the diagnosis is delayed by 24 h, the mortality rate increases eight-fold [44].

**Figure 6:**
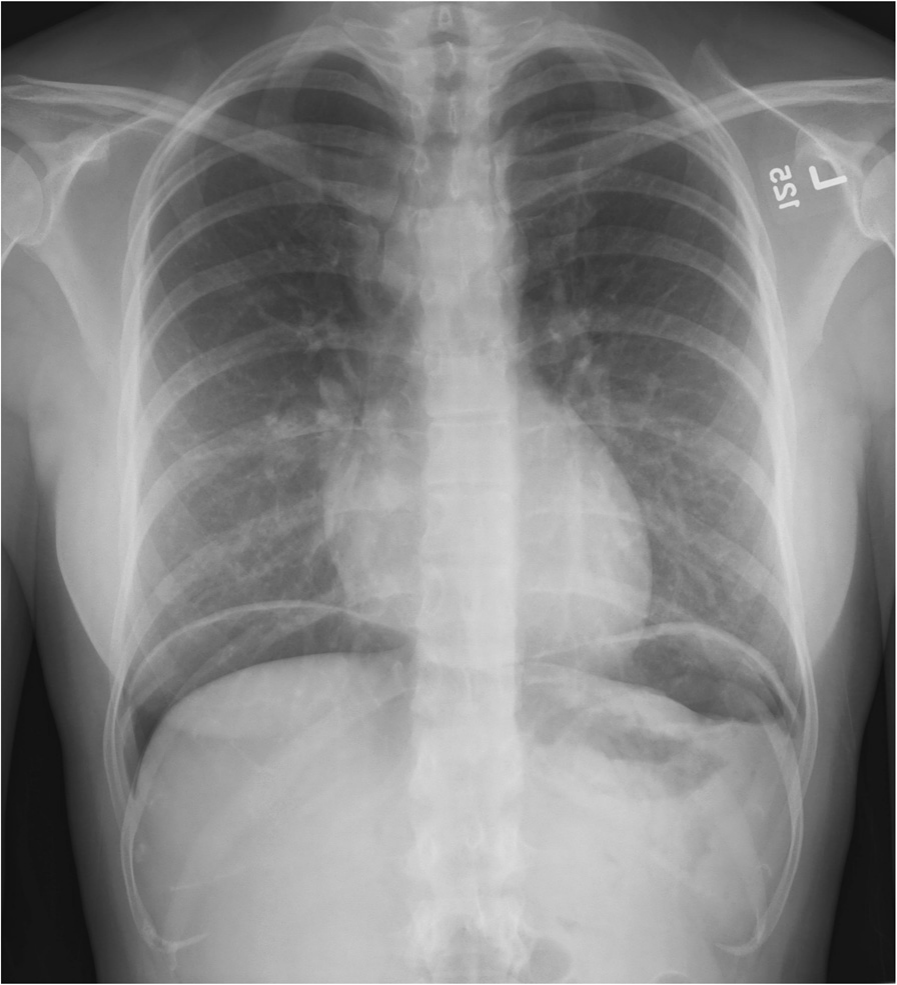
Upright chest film showing free air under the diaphragm.

Lack of abdominal pain is not the only atypical presentation seen in the elderly. The most common presenting sign is melena [41]. Due to physiologic changes including decreased abdominal musculature, rigidity is absent in approximately 80% of elderly patients who present with perforated PUD, and free air is appreciated on only about 40% of plain radiographs [37] (Figure 6). Vital signs may be normal [21]. New-onset congestive heart failure from chronic anemia has been reported [40].

In addition to the changing physiology of the elderly patient, the increased use of medications such as nonsteroidal anti-inflammatory drugs (NSAIDs), aspirin, steroids, and anticoagulants contribute to an increasing incidence of PUD [40]. Up to 40% of elderly patients take an NSAID, and it has been shown that age is an independent risk factor for gastroduodenal injury. Moreover, the incidence of *Helicobacter pylori* ranges from 53% to 73% in this population, contributing to an increased risk of duodenal ulcers [40],[46].

##### Biliary disease and pancreatitis

Biliary disease, specifically acute cholecystitis (AC), is the leading surgical emergency among the elderly [47]. The reasons are multifold: age-related changes in the vasculature, increased comorbidities, and an increased incidence of gallstones. The diagnosis might not be straightforward in the elderly. Furthermore, the risk of complications related to AC increases in this population [48].

**Figure 7:**
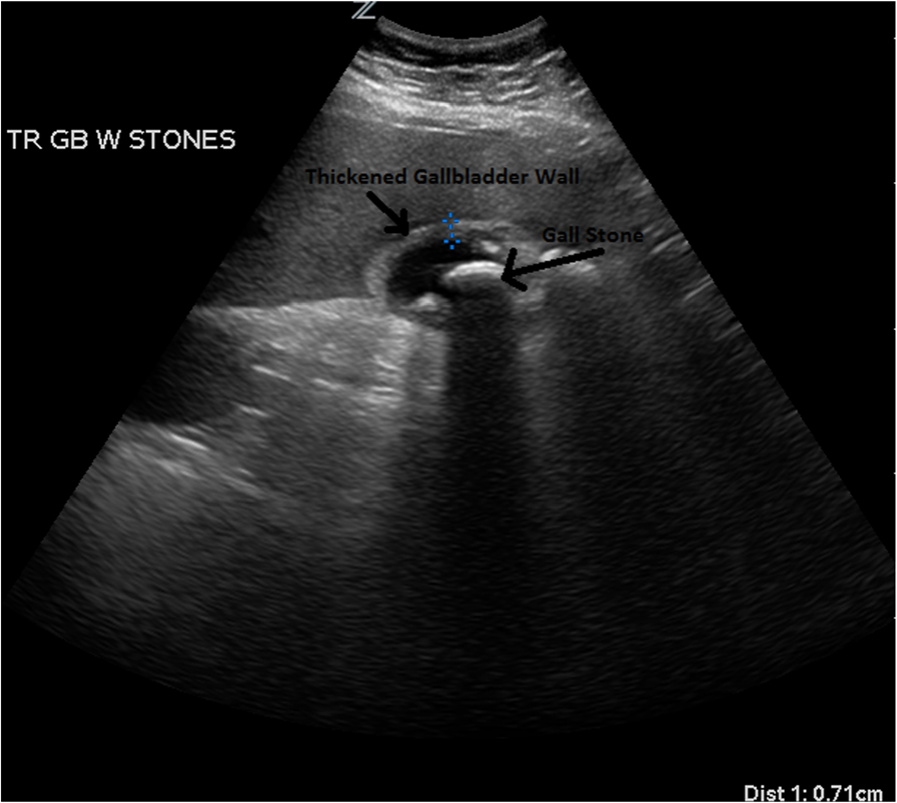
**Ultrasound of a patient with acute cholecystitis.** A very large gallstone with significant surrounding edema can be seen.

The typical presentation of AC is a female patient in her forties with fever, right upper quadrant pain, nausea, and vomiting. Elderly patients often do not have these symptoms. Although they might have the classic right upper quadrant pain, nearly 40% do not have nausea and vomiting, and many are afebrile. In addition, laboratory tests that yield abnormalities indicative of AC, such as leukocytosis and abnormal liver function tests, could be normal [49]. Ultrasound, the initial diagnostic study of choice, has good sensitivity and specificity in the elderly [50] (Figure 7).

**Figure 8:**
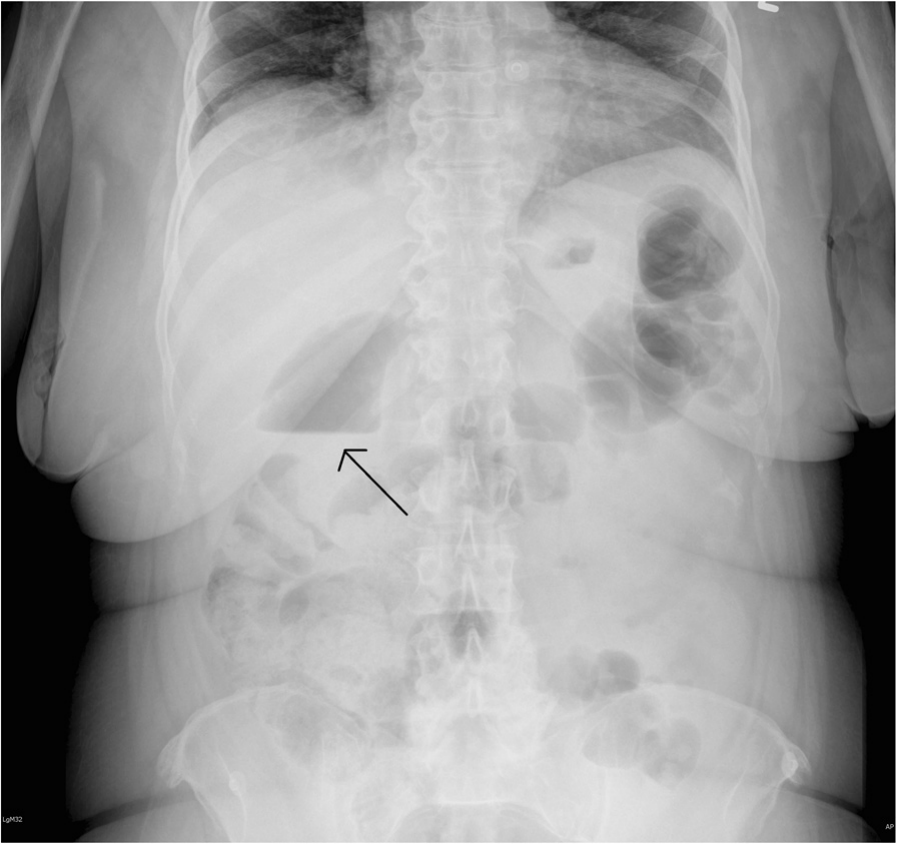
Upright abdominal radiograph demonstrating an air-fluid level in the gallbladder, diagnostic for emphysematous cholecystitis.

Complications of cholecystitis such as choledocholithiasis, cholangitis, and emphysematous cholecystitis are also much more common in the elderly [48]. Due to the poor vascularity of the gallbladder, the elderly are at increased risk of perforation and emphysematous cholecystitis [51] (Figure 8). It is important to consider these complications and act expeditiously. The administration of broad-spectrum antibiotics with anaerobic coverage is recommended, as well as early surgical consult. Delayed surgical management can increase morbidity and mortality rates unnecessarily [52].

The incidence of pancreatitis increases 200-fold after age 65 [53]. Pancreatitis often presents typically in the old as well as the young, with ‘boring’ epigastric pain radiating into the back, associated with vomiting. However, some elderly patients with pancreatitis present with only hypotension and altered mental status, which broadens the differential greatly [39]. In those more than 80 years old, the risk of necrotizing pancreatitis increases significantly. Other diagnoses, such as mesenteric ischemia, may present with elevated amylase as well. Consider CT scanning early in elderly patients with suspected pancreatitis if the diagnosis is in doubt or alternative diagnoses are being considered.

#### 1.1.4 Non-abdominal causes of abdominal pain

Failing to consider extra-abdominal causes in the patient presenting with abdominal pain is a frequent pitfall. Several life-threatening illnesses can present with abdominal pain only.

Myocardial infarction is the most important diagnosis to consider. One-third of women above the age of 65 who have an acute myocardial infarction present with only abdominal pain. This is most common in diabetics and in patients with inferior infarctions [54]. In a study of elderly patients with unstable angina, 45% did not have any chest pain, 8% had epigastric pain, 38% had nausea, and 11% had vomiting [55]. Patients with atypical presentations tend to have longer delays in treatment and therefore an increased mortality rate [54]. Therefore, it is prudent to obtain an electrocardiogram in every elderly patient with epigastric pain. Other cardiac illnesses that can present with abdominal pain are congestive heart failure and pericarditis.

Pulmonary processes, especially those involving the lower lobes, are another cause of abdominal pain. These include pneumonia, pulmonary embolism, pleural effusion, and pneumothorax. Metabolic causes such as diabetic ketoacidosis (DKA), hypercalcemia, Addisonian crisis, and porphyria should be considered as well in the appropriate clinical circumstances. Herpes zoster should be considered in patients with well-localized abdominal pain. It can be very difficult to diagnose in the pre-vesicular phase.

Genitourinary issues are a significant source of abdominal pain. Cystitis and pyelonephritis often are associated with abdominal pain. Pyelonephritis can present with only abdominal pain or vomiting without any urinary symptoms [54]. A particularly challenging entity to diagnose correctly (and therefore treat) is prostatitis. Both acute and chronic prostatitis require a significantly longer course of antibiotics than other urinary tract infections [56].

Asymptomatic bacteriuria affects a significant number of elderly patients - women more than men and institutionalized patients more than community dwellers [56]. However, acute abdominal pain should not be attributed to asymptomatic bacteriuria. Acute urinary retention is another diagnosis that should be entertained and can easily be missed in patients who are unable to provide a clear history. It might be caused by a urinary tract infection, a stone, or medications, usually in the setting of an enlarged prostate.

## 2 Conclusions

Elderly patients with acute abdominal pain present a significant challenge to even the most seasoned clinician (Table 3). The atypical presentation of disease is distinctly typical in this group. Despite seemingly innocuous symptoms, many elderly patients with acute abdominal pain have serious pathology, including surgical disease and extra-abdominal processes manifesting with abdominal complaints. The wary clinician will approach these patients with a broad differential and a logical, step-wise approach to ensure that all possibilities are considered in a timely fashion.

**Table 3:** Pitfalls in the evaluation of abdominal pain in the elderly

	**Pitfalls**
1.	Relying on normal laboratory results to rule out AMI.
2.	Misdiagnosing AMI as gastroenteritis.
3.	Relying too heavily on classic presentations of common illnesses in the elderly.
4.	Over-reliance on a positive urinalysis as indicating the cause of acute abdominal pain.
5.	Relying on classic findings and history to rule out appendicitis.
6.	Expecting abdominal rigidity when considering a visceral perforation.

## Competing interests

The authors declare that they have no competing interests.

## Authors’ contributions

RS wrote several sections of this manuscript as well as organized, edited, and prepared the final submission. TP contributed several sections of the manuscript, edited, and primarily organized the literature sources used in the paper as well as approved the final submission. DK contributed several sections of the manuscript and edited and approved the final submission. JM wrote the introduction and conclusion, provided experience and insight regarding the content, provided editorial revisions and images, and approved the final submission. All authors read and approved the final manuscript.
